# Mycoviruses as Biocontrol Agents: Potential Applications for Managing Pests and Diseases of Oilseed Rape ( *Brassica napus* )

**DOI:** 10.1111/ppl.71077

**Published:** 2026-08-22

**Authors:** Harry J. Aston, Robert H. A. Coutts, Yong‐Ju Huang, Ioly Kotta‐Loizou

**Affiliations:** ^1^ School of Health, Medicine and Life Sciences, University of Hertfordshire Hatfield UK; ^2^ Department of Life Sciences Faculty of Natural Sciences, Imperial College London London UK; ^3^ Division of Evolution, Infection and Genomics, School of Biological Sciences Faculty of Biology, Medicine and Health, University of Manchester Manchester UK

**Keywords:** biocontrol, endophyte, hypervirulence, hypovirulence, mycoviruses

## Abstract

Oilseed rape ( *Brassica napus* ) production faces a wide range of biotic threats. Pests typically pose a greater threat than diseases to oilseed rape, yet both vary considerably across the major cultivating regions. Management strategies often rely heavily on the use of chemical pesticides which may have harmful effects on the environment, thus developing new forms of biological control is key to future oilseed rape security. Mycoviruses are viruses that infect fungi, altering phenotypes of their host upon infection and are emerging as potential biocontrol agents. In plant pathogenic fungi, mycovirus‐induced hypovirulence can be used to reduce disease severity and in entomopathogenic fungi (EPF), mycovirus‐induced hypervirulence can enhance effectiveness of EPF‐based pest control. Mycoviruses of some pathogenic fungi, like *Sclerotinia sclerotiorum*, have been explored extensively and present many candidates for biocontrol. Viral diversity of other pathogenic fungi remains poorly understood and therefore presents an opportunity for new discoveries. In this review, we summarise existing mycoviruses of pathogenic fungi relevant to oilseed rape, highlighting those species with potential to control their respective pests/diseases. Furthermore, recent advancements in mycovirus research and the current challenges facing mycovirus‐based applications are also discussed.

## 1 Introduction

### 1.1 Pests and Diseases of Oilseed Rape

**FIGURE 1:**
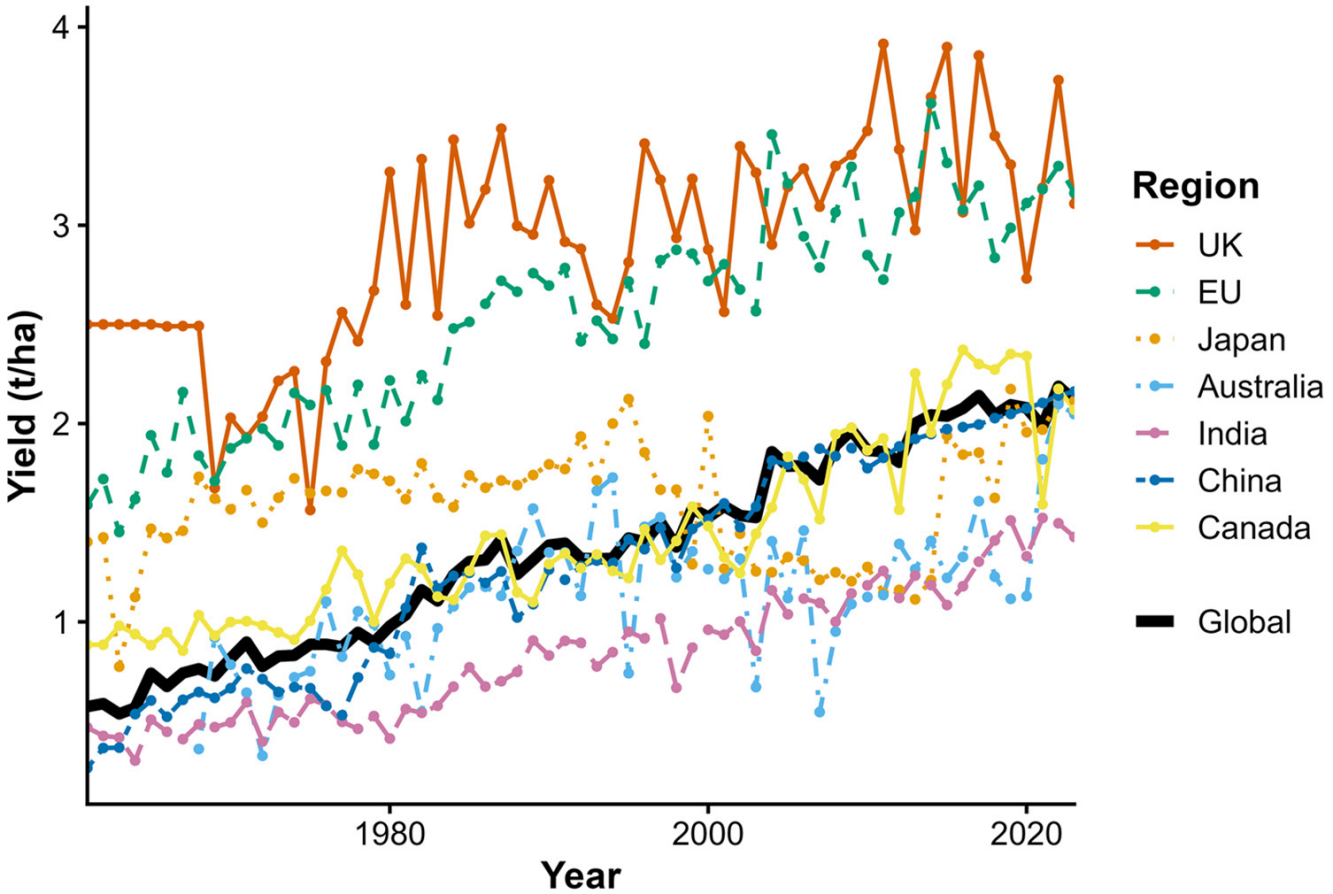
Oilseed rape yields per region since 1960. Average annual yields of oilseed rape in major producing regions compared to the global average (black; FAO database).

Oilseed rape ( *Brassica napus* ) or canola, is an important global crop and a member of the *Brassicaceae* family. *Brassicaceae* consists of 354 genera and around 4000 species, including many other economically and scientifically significant species (POWO 2026). Oilseed rape is mostly cultivated in temperate regions for vegetable oil and animal feed production, plays a crucial role in cereal crop rotation cycles and remains a vital part of many agricultural economies. Throughout the 20th century oilseed rape production increased stably, yet since the 1990s yields have plateaued in some regions (Figure 1; FAO database). Poor cultivar choice, high cropping efficiency and persistent biotic pressures are among many factors contributing to the plateau. In Europe, the 2014 neonicotinoid ban is thought to have significantly contributed to the fall in oilseed rape production (Scott and Bilsborrow 2019). Improper use of alternative chemical pesticides, such as pyrethroids, has led to emergence of resistant strains and has a detrimental pollutive impact on the environment (Heimbach and Müller 2013; Antwi and Reddy 2015; Das et al. 2016). Elevated temperatures negatively impact oilseed rape production; thus, current climatic trends jeopardise future oilseed rape security (Kutcher et al. 2010). Research on and subsequent use of resistant cultivars has improved productivity of most crops. However, low genetic diversity of the *B. napus* genome, due to its allotetraploid origin from *B. rapa* and *B. oleracea* , provides limited natural resistance to pathogens (Liu et al. 2014a; Rahman 2013). Thus, compared to similarly prevalent crops, oilseed rape is particularly vulnerable to biotic stresses.

A wide variety of diseases and pests considerably reduce oilseed rape and other brassicaceous yields (Table 1; Zheng et al. 2020). Notably, the fungi *Sclerotinia sclerotiorum* (sclerotinia stem rot) and *Leptosphaeria maculans* and *L. biglobosa* (anamorph *Phoma lingam*; phoma stem canker) and the protist *Plasmodiophora brassicae* (clubroot) persist as the most prominent diseases across all major regions of cultivation. *B. napus* also hosts generalist fungal pathogens like *Alternaria* spp., *Botrytis cinerea, Rhizoctonia solani* and *Fusarium* spp. Generalist pathogens have less direct impact on oilseed rape yields, yet their relevance in general disease management, particularly biological solutions, remains highly important (Deng et al. 2022). Pests are animals that damage the host through feeding and are globally considered more threatening to oilseed rape than diseases (with the exception of Australia) yet vary considerably within regions. Current integrated pest management (IPM) strategies for oilseed rape pests and diseases combine surveillance and monitoring, cultural practices, resistant cultivars and chemical controls. Novel biological control alternatives, such as parasitic wasps, beneficial microbes and double‐stranded (ds)RNA applications vary in effectiveness under field conditions and often lack commercial viability. Biocontrol agents (BCAs) can be very effective in conjunction with established IPM strategies but are currently insufficient to protect crops on their own (Agriculture and Horticulture Development Board; AHDB 2026). Thus, alternative sustainable forms of disease/pest control must be developed for future oilseed rape security.

**TABLE 1:** Major threats to oilseed rape cultivation across the producing regions.

Country	Oilseed rape production (2024–25) in million tons	Diseases									
		Sclerotinia stem rot (*Sclerotinia sclerotiorum*)	Phoma stem canker (*Leptosphaeria biglobosa and L. maculans*)	Clubroot (*Plasmodiphora brassicae*)	Alternaria blight (*Alternaria* spp.)	Grey mould (*Botrytis cinerea*)	Verticillium stem stripe (*Verticillium longisporum*)	Downy mildew (*Peronospora parasitica*)	Powdery mildew (*Erysiphe cruciferarum*)	Light leaf spot (*Pyrenopeziza brassicae*)	White leaf spot (*Pseudocercosporella capsellae*)
Canada	19.19	+		+							
China	15.80	+						+			
India	11.52	+			+						
Australia	5.44	+	+					+			+
France	4.63	+	+								
Germany	3.99	+		+			+				
Poland	3.64			+			+				
Ukraine	3.10	+	+		+			+	+		+
Japan	3.00	+		+							
Romania	2.49	+	+		+				+		
Czechia	1.02	+	+		+	+					
UK	0.90	+	+	+			+			+	
Global	85.65										

Country	Pests									References
	Cabbage stem flea beetle (*Psylliodes chrysocephala*)	Flea beetles (*Phyllotetra* spp.)	Aphids (e.g., *Brevicoryne brassicae, Myzus persicae*)	Weevils (*Ceuthorrhynchus* spp.)	Diamondback moths (*Plutella xylostella*)	Pollen beetles ( *Brassicogethes aeneus* )	Armyworms (*Mamestra* spp.)	Slugs (*Deroceras* spp.)	Butterflies/caterpillar**s**	
Canada	+							+		PPMN (2026) and Zheng et al. (2020)
China	+			+	+	+				Zheng et al. (2020)
India			+						+	Meena et al. (2010), and Yadav and Rathee (2020)
Australia						+				Murray and Brennan (2012) and Arthey (2020)
France	+			+	+					Inovia (2019)
Germany	+			+	+		+			Arthey (2020)
Poland				+	+		+			Arthey (2020)
Ukraine				+	+	+				Zheng et al. (2020), Tsytsiura et al. (2023) and Stankevych et al. (2020)
Japan			+				+		+	Nakui and Mikami (2025)
Romania		+	+	+						Zală et al. (2023)
Czechia	+	+		+						Vykydalová et al. (2024) and Zheng et al. (2020)
UK	+		+	+		+				Pest and Disease Survey (2025) and AHDB (2026)
Global										

*Note:* Oilseed rape production during the 2024/25 growing season in Europe (Agridata database), Australia (AOF 2024) and other major producing regions (FAO database). Significant pests and diseases threatening oilseed rape production are summarised for each region.

### 1.2 Mycoviruses

Mycoviruses or fungal viruses, infect fungi across diverse ecological contexts (Yu et al. 2010; Kotta‐Loizou et al. 2015; Nerva et al. 2019; Battersby et al. 2024). Almost all mycoviruses have RNA genomes and are currently characterised into 45 families (https://ictv.global/ accessed on 12th January 2026), many of which are shared with closely related plant viruses. Improvements in next generation sequencing (NGS) and frequency of mycovirus screening studies have led to an increasing abundance of novel mycoviruses discovered in recent years (Ruiz‐Padilla et al. 2021; Kondo et al. 2022). Unlike plant and animal viruses, mycoviruses typically lack an extracellular phase of their life cycle. Mycovirus transmission occurs vertically, via spores or horizontally, through anastomosis, but host specificity is less well understood (Buivydaitė et al. 2024). Host specificity can be generally confined to vegetatively compatible strains or closely related species, yet cross‐genus and even cross‐kingdom mycovirus infections have been reported (Deng et al. 2022; Dai et al. 2024).

### 1.3 Mycovirus‐Based Biocontrol Applications

Effective mycovirus‐based control relies on a mycovirus infection inducing phenotypic changes in a fungal host. Despite most infections being asymptomatic, many induced phenotypes can be used to manipulate pathogenic fungi. Changes to host growth, virulence, sporulation, pigmentation (Shah et al. 2018), mycotoxin production (Filippou et al. 2025), antimicrobial resistance (Sass, Kotta‐Loizou, et al. 2023) and stress tolerance (Sass, Martinez, et al. 2023) have all been associated with mycovirus infections and are often accompanied by significant host transcriptomic changes (Shi et al. 2025).

Mycovirus‐based disease control was first exploited in chestnut blight (Van Alfen et al. 1975). The mycovirus Cryphonectria hypovirus 1 (CHV1) reduced virulence (hypovirulence) in *Cryphonectria parasitica* . Hypovirulent CHV1‐infected spores were released and reduced disease incidences across Europe. As most mycoviruses cannot exist extracellularly, virus‐infected spores are, at present, the most promising method for application. Fungal spores are pigmented with melanin, importantly protecting fungal (and mycoviral) nucleic acids from unwanted UV‐induced mutations and can withstand harsh conditions for long periods (Pacelli et al. 2017). Consistent with their non‐lethal nature, mycoviruses require host machinery to replicate and are thus incapable of completely abolishing diseases on their own. Mycovirus‐mediated increase in antifungal sensitivity is therefore a particularly valuable trait when considering the integration of mycovirus‐based applications alongside existing fungicide treatments.

Entomopathogenic fungi (EPF) infect insects, colonise plants endophytically and are a commercial pest control strategy. Mycoviruses can also infect and alter EPF phenotypes, enhancing biocontrol potential (Kotta‐Loizou 2021). Desirable mycovirus‐induced traits in EPFs include increased virulence (hypervirulence), growth, sporulation, mycotoxin production, stress tolerance and reduced antifungal sensitivity and may enhance EPF colonisation during both their entomopathogenic and endophytic stages.

Recent advancements in dsRNA‐based applications in agriculture elevate the potential of many RNA mycoviruses as BCAs (Vatanparast et al. 2024). However, as several single‐stranded (ss)DNA mycoviruses can exist extracellularly (Yu et al. 2010; Khalifa and MacDiarmid 2021), DNA mycovirus applications arguably show more promise.

This review attempts to provide an up‐to‐date summary of mycoviral research relevant to the pests and diseases impacting oilseed rape plants and other brassicas. Mycoviruses known to infect each pathogenic fungus are analyzed for potential biocontrol suitability and the current advantages/limitations of mycovirus‐based applications are discussed.

## 2 Mycoviruses Associated With the Diseases of Oilseed Rape: Plant Pathogenic Fungi

### 2.1 Sclerotinia Stem Rot

Sclerotinia stem rot (SSR) is caused by the ascomycete *Sclerotinia sclerotiorum* (family *Sclerotiniaceae*). *S. sclerotiorum* infects over 700 plant species and is widely regarded as both a generalist plant pathogen (Derbyshire et al. 2022) and a high impact pathogen of oilseed rape (Zheng et al. 2020). Compact melanised hyphal masses known as sclerotia survive long periods under harsh conditions, germinate producing ascospores and begin infections as small lesions on *B. napus* leaves/petals (Brodal and Asdal 2021). Infections begin in the petals and spread down into the petioles, leaves and stems. Infections produce white mycelia on the stem surface, black sclerotia inside the stem and result in stem lesions and rotting, thus reducing seed yields (Ding et al. 2021). Prevalence and severity of SSR varies with climatic conditions (Wójtowicz and Wójtowicz 2023), yet *S. sclerotiorum* may account for up to 80% of the total oilseed rape yield losses (Yang et al. 2018). Fungicides can be effective in controlling SSR in oilseed rape (Zamani‐Noor 2021). However, progress of research into natural resistance to SSR is slow, largely due to the complexity of both *S. sclerotiorum* pathogenicity and *B. napus* genetic resistance (Ding et al. 2021).

Due to the cosmopolitan nature and wide host range of this necrotrophic pathogen, large swathes of research into the viruses of *S. sclerotiorum* already exist. Currently, 12 out of the 22 mycovirus families found to infect *S. sclerotiorum* harbour viral candidates for SSR control (Table 2). One of the most well‐researched mycoviruses to date, Sclerotinia sclerotiorum hypovirulence‐associated DNA virus (SsHADV‐1; family: *Genomoviridae*), infects and induces hypovirulence in its native host *S. sclerotiorum* (Yu et al. 2010). SsHADV‐1 was the first ssDNA mycovirus discovered, is unusually capable of both intracellular and extracellular transmission and remains the most promising candidate for mycovirus‐based control of SSR.

**TABLE 2:** Mycoviruses of plant pathogenic and entomopathogenic fungi with potential for biocontrol application relevant to oilseed rape.

Pathogen host	Virus family	Virus	Virus‐mediated phenotypes^a^	References
*Sclerotinia sclerotiorum*	*Alphaflexiviridae*	SsDRV	↓ Virulence on oilseed rape	Xie et al. (2006)
	*Deltaflexiviridae*	SsDFV2	↓ Growth ↓ Sclerogenesis ↑ Transmissibility (regardless of VIGs) ↓ Virulence (lesions on oilseed rape)	Hamid et al. (2018)
	*Endornaviridae*	SsEV3	↓ Growth ↓ Virulence (lesions on oilseed rape)	Mu et al. (2021)
			↓ Sclerogenesis ↓ Resistance to abiotic stresses ↓ Infection cushion formation (oilseed rape, soybean)	Mu et al. (2025)
	*Genomoviridae*	SsHADV‐1	↓ Virulence (disease severity on oilseed rape) ↓ Growth ↓ Sclerogenesis ↑ Transmissibility (regardless of VIGs)	Yu et al. (2010)
			↑ Oilseed rape growth ↑ Oilseed rape resistance to Sclerotinia stem rot	Zhang et al. (2020)
			↑ Cereal resistance to Fusarium head blight, stripe rust, & rice blast	Tian et al. (2020)
			↑ Dry bean resistance to Sclerotinia stem rot, grey mould, & root rot	Fu et al. (2024)
			↑ Wheat microbial diversity & N‐content	Tian et al. (2023)
	*Hypoviridae*	SsHV2‐L	Delayed maturation of sclerotia ↓ Virulence (lesions on lettuce & soybean)	Marzano et al. (2015)
			↓ Growth & pigmentation	Mochama et al. (2018)
	*Mitoviridae*	SsMV1	↓ Growth & sclerogenesis (SsMV1/SsMV2 co‐infection) ↓ Virulence (lesions on lettuce & soybean)	Xie and Ghabrial (2012)
			Abnormal hyphal tip morphology & mitochondrial malformation	Xu et al. (2015)
		SsMV2	↓ Growth & virulence (lesions on tomato leaves)	Khalifa et al. (2022)
	*Mymonaviridae*	SsNSRV‐1	↓ Growth, no scleriogenesis & abnormal hyphal tip morphology No pathogenicity on oilseed rape	Liu et al. (2014b)
	*Narnaviridae*	SsNaV5	↓ Virulence (lesions on oilseed rape), growth & pigmentation	Hai et al. (2022)
	*Partitiviridae*	SsPV1	↓ Virulence (lesions on *A. thaliana* ) and growth Severely damaged organelles ↑ Transmissibility (regardless of VIGs)	Xiao et al. (2014)
	*Reoviridae*	SsMYRV4	↓ Virulence (lesions on oilseed rape), growth and sclerogenesis ↑ Self and heterologous virus transmissibility on oilseed rape (suppression of non‐self recognition)	Wu et al. (2017)
	*Shuviridae* (proposed family)	SsAShV1	↓ Virulence (lesions on oilseed rape) ↑ Antifungal volatile compounds and microbial competition	Liu et al. (2026)
	*Secoviridae*	HuSRV1	↓ Virulence (lesions on oilseed rape) & growth	Azhar et al. (2019)
	*Yadokaviridae*	SsYkV1	Hijacking heterologous viral capsids to facilitate self‐replication	Jia et al. (2022)
*Leptosphaeria biglobosa*	*Botybirnaviridae*	LbBV‐1	Asymptomatic in natural host *L. biglobosa* ↓ Virulence and reduced sclerogenesis in *B. cinerea* on *Nicotiana benthamiana* ↑ Cross‐genus transmission *in planta*	Deng et al. (2022)
	*Quadriviridae*	LbQV‐1	↑ Virulence (lesions on oilseed rape), growth, sporulation and pigmentation ↓ Virulence of subsequent *L. maculans* attacks (lesions on oilseed rape)	Shah et al. (2018)
			↑ Systemic resistance against *L. maculans* in oilseed rape	Shah et al. (2020)
*Botrytis cinerea*	*Endornaviridae*	BcEV4	↓ Growth and virulence (lesions on strawberry)	Ahmed et al. (2023)
	*Genomoviridae*	BGDaV1	↓ Growth and virulence (lesions on oilseed rape) ↑ Local resistance against future *B. cinerea* infection (lesions on oilseed rape)	Khalifa and MacDiarmid (2021)
	*Hypoviridae*	BcHV1	↓ Virulence (reduced lesions on oilseed rape & tomato) Inhibition of infection cushion formation	Hao et al. (2018)
	*Mitoviridae*	BcMV1	↓ Growth & virulence (lesions on oilseed rape) Mitochondrial malformation	Wu et al. (2010)
		BcMV9	↓ Growth & virulence (lesions on tomato)	Duan et al. (2024)
		BcMV10	↓ Growth & virulence (lesions on tobacco, tomato, apple & *Paris polyphylla*)	Wang et al. (2022)
	*Partitiviridae*	SsPV1	↓ Growth & virulence (lesions on *A. thaliana* ) ↑ Conidiation	Xiao et al. (2014)
		BcPV2	Defective conidiation & sclerogenesis ↓ Virulence (lesions on oilseed rape, tomato, tobacco, cucumber, strawberry, apple & grape)	Kamaruzzaman et al. (2019)
			↑ Outcompetes virulent *B. cinerea* /*S. sclerotiorum* isolates	Kamaruzzaman et al. (2020)
			↑ Stimulates growth in tomato	Kamaruzzaman et al. (2021)
*Alternaria* spp.	*Alternaviridae*	AaV‐1	↓ Growth & mycelial collapse ( *A. alternata* )	Aoki et al. (2009)
	*Botybirnaviridae*	AaBRV1	↓ Growth & antifungal sensitivity ( *A. alternata* ) Altered pigmentation	Zhang et al. (2025)
	*Chrysoviridae*	AaCV1	↓ Growth ( *A. alternata* ) ↑ Mycotoxin production & virulence (lesions of *A. alternata* on *Pyrus pyrifolia* )	Okada et al. (2018)
			↓ Growth & mycotoxin production ( *A. alternata* ) ↓ Sporulation ( *A. tenuissima* ) ↑ Antifungal sensitivity ( *A. tenuissima* )	Li et al. (2022)
	*Hypoviridae*	AaHV1	↓ Growth & virulence (lesions of *A. alternata* & *B. dothidea* on apple)	Li et al. (2019)
	*Mitoviridae*	AaMV1	↑ Transmissibility of AaCV1 ( *A. alternata* )	Li et al. (2022)
	*Totiviridae*	AaVV1‐QRH	↓ Growth, virulence (lesions of *A. alternata* & *A. gomphrenae* on tobacco) Deformed colony morphology	Wang et al. (2025)
*Beauveria bassiana*	*Amalgaviridae*	BbNV‐1	↑ Virulence (↑ mortality of *Galleria mellonella;* BbPmV‐1/BbNV‐1 co‐infection)	Kotta‐Loizou and Coutts (2017)
	*Chrysoviridae*	BbCV‐2	↓ Virulence (↓ mortality of *Ostrinia furnacalis*) Extracellular transmission	Zhang et al. (2023)
			↑ Growth & conidiation ↑ Microbial competition (↑ inhibition of *S. sclerotiorum* & *B. cinerea* )	Sui et al. (2024)
	*Polymycoviridae*	BbPmV‐1	↑ Virulence (↑ mortality of *G*. *mellonella*; BbPmV‐1/BbNV‐1 co‐infection)	Kotta‐Loizou and Coutts (2017)
			↑ Growth, conidiation & pigmentation (BbPmV‐1/−3 co‐infection)	Filippou et al. (2021)
			↑ Mycotoxin production in *T. molitor* (BbPmV‐1/BbNV‐1 co‐infection)	Filippou et al. (2025)
			↑ Virulence (↑ mortality of *Galleria mellonella*; BbPmV‐1/BbPV‐2 co‐infection) ↑ Osmotic, heat & UV tolerance ↑ Microbial competition (↑ inhibition of *T. harzianum*)	Rueda‐Maíllo et al. (2025)
		BbPmV‐3	↑ Growth, conidiation & pigmentation (BbPmV‐1/−3 co‐infection)	Filippou et al. (2021)
		BbPmV‐4	↓ Conidiation ↑ Virulence (↑ mortality of *O. furnacalis*)	Kang et al. (2023)
		BbPmV‐4‐2	↑ Sporulation ↑ Heat & UV tolerance ↑ Virulence (↑ mortality of *Galleria mellonella*)	Shi et al. (2025)
	Unclassified	BbOCuV1	↑ Growth, sporulation and biomass ↑ Plant resistance against *S. sclerotiorum* and *Fusarium asiaticum*	Guo et al. (2026)
*Metarhizium* spp.	*Partitiviridae*	MfPV1	↑ Conidiation ↑ Virulence (*M. flavoviride, M. anisopliae* & *M. pingshaense*; ↑ mortality of *Plutella xylostella* & *Spodoptera frugiperda* )	Guo et al. (2024)
		MaV‐RJ	↑ Conidiation ↓ Endochitinase production (*M. anisopliae*)	De La Paz Giménez‐Pecci et al. (2002)
		MmPV‐1	↓ Conidiation ↓ Heat & UV tolerance ↑ Inhibition of antiviral compounds ( *M. majus* )	Wang, Yang, Shi, et al. (2023)
	*Polymycoviridae*	MaPmV1	↑ Growth & conidiation ↓ UV tolerance	Wang, Yang, Lu, and Huang (2023)

^a^ Only mycovirus infections causally linked (not correlated) to phenotypic infections are summarised in this table.

First discovered in the *S. sclerotiorum* strain DT‐8, SsHADV‐1 infections display reductions in growth, virulence and smaller, delayed production of sclerotia, whilst growing on *B. napus* . SsHADV‐1‐mediated hypovirulence was transmitted into vegetatively incompatible *S. sclerotiorum* strains and closely related species of the same genus, *S. minor* and *S. nivalis* (Yu et al. 2010, 2013). Overcoming vegetative incompatibility barriers in fungal populations is an important characteristic for an effective fungal‐based BCA.

SsHADV‐1's ability to modulate its host's environment has been thoroughly investigated. Genes of key metabolic pathways, synthesis of secondary metabolites, membrane formation and G‐protein activity were all differentially regulated, suggesting SsHADV‐1 alters intracellular signalling pathways during early‐stage infection (Ding et al. 2019). Furthermore, downregulation of host antiviral silencing genes likely accommodates SsHADV‐1 replication and reduced expression of virulence factors lowers virulence (Zhang et al. 2020). SsHADV‐1‐mediated transcriptional modulations enable DT‐8 to grow endophytically, unlike its uninfected *S. sclerotiorum* counterpart and enter a symbiotic relationship with *B. napus* (Qu, Zhang, et al. 2021).

The *B. napus* transcriptome is also altered by SsHADV‐1‐infected *S. sclerotiorum*. Upregulation of SWEET sugar transporters, plant cell wall degrading enzymes and genes known to positively regulate detoxification of fungal effectors may encourage DT‐8 colonisation of plant tissue (Qu, Fu, et al. 2021). Inoculating *B. napus* seeds with DT‐8 improved abundance of plant‐associated microbes, provided protection against further SSR infection and increased yields under field conditions (Qu et al. 2020). Furthermore, spraying DT‐8 hyphal suspensions onto *B. napus* during the early blossom stage reduced SSR severity by 30.1%–67.7%, thus increasing yield by 6.9%–14.9% (Zhang et al. 2020).

DT‐8 strengthens microbial communities, upregulates plant immune pathways and reduces disease severity in a range of other important crops (Qu, Zhang, et al. 2021; Tian et al. 2020; Fu et al. 2024). Abundance of plant growth‐promoting rhizobacteria and other BCAs in the rhizosphere of DT‐8 inoculated wheat were increased (Tian et al. 2023). Many of these microbial genera contain species associated with induced systemic resistance (ISR). ISR provides wide‐scale resistance against a broad range of pathogens: a powerful characteristic in disease control. DT‐8 provided heightened protection against *Fusarium* head blight, stripe rust and rice blast in a variety of cereals. Treatment of wheat seeds with DT‐8 increased growth by 4%–18% and consistently reduced severity of *Fusarium* head blight between 40% and 60% (Tian et al. 2020). Fu et al. (2024) also demonstrated DT‐8's ability to protect the legume *Phaseolus vulgaris* or common bean, against *S. sclerotiorum*, *B. cinerea* and *R. solani* . Thus, the ability of SsHADV‐1‐infected *S. sclerotiorum* strains to bio‐prime oilseed rape and many other important crops has been widely proven and the current focus should be on adapting SsHADV‐1 infected strains for commercial applications.

Mycovirome screening studies have begun to uncover the prevalence of SsHADV‐1 (Kraberger et al. 2013) and other *S. sclerotiorum* viruses in the environment (Mu et al. 2017). For example, 68 mycoviruses were identified from screening S. *sclerotiorum* isolates from a single field over three years (Jia et al. 2021). Screening of a hypovirulent *S. sclerotiorum* isolate also revealed nine viruses co‐infecting a single isolate (Mu et al. 2021). Prevalence of SsHADV‐1 in insect populations uncovered its ability to replicate in unconventional insect hosts. SsHADV‐1 can replicate in two evolutionary diverse insects: *Lycoriella ingenua*, a mycophagous pest of mushroom cultivation; and the fall armyworm *Spodoptera frugiperda* (Liu et al. 2016). Armyworms, especially the Bertha armyworm, cause significant oilseed rape losses in Canada (Erlandson et al. 2019). SsHADV‐1 was exchanged between fungus and insect during feeding and transmitted vertically into larval offspring (Liu et al. 2016). SsHADV‐1 DNA was also found in damselfly cells. Interestingly, DT‐8‐infected damselflies laid more eggs compared to virus‐free damselflies (Dayaram et al. 2015). In choice experiments, damselflies preferred feeding on SsHADV‐1‐infected mycelia due to volatile compounds produced by SsHADV‐1‐infected *S. sclerotiorum* (Liu et al. 2016). Thus, SsHADV‐1 appears to facilitate a mutually beneficial relationship between *S. sclerotiorum* and several insect hosts. A virus's ability to replicate and transmit through insect vectors is a valuable characteristic for enhancing transmission through a fungal population. However, cross‐genus and cross‐kingdom infections and the presence of SsHADV‐1 in environmental moulds, which can also infect common foods (Fu et al. 2022), should warrant further investigation.

*S. sclerotiorum* hosts mycoviruses from many other families with potential to control SSR (Table 2). Hypoviruses (family *Hypoviridae*, capsidless monosegmented ssRNA viruses) have been discovered to infect several hypovirulent *S. sclerotirum* strains (Xie et al. 2011; Hu et al. 2014; Mu et al. 2021; Luo et al. 2022). Characterised in 2015, a strain of Sclerotinia sclerotiorum hypovirus 2 (SsHV2) known as SsHV2‐Lactuca (SsHV2‐L) induced hypovirulence together with the co‐infecting Sclerotinia sclerotiorum endornavirus 1 (SsEV1) (Marzano et al. 2015). SsHV2‐L was found to reduce growth, sclerotia production and lesions on soybean ( *Glycine max* ) and lettuce ( *Lactuca sativa* ; Mochama et al. 2018). Interestingly, SsHV2 is known to infect and increase growth of another pathogenic fungus, *Monilinia fructicola* or brown rot, alongside two other co‐infecting mycoviruses (Tran et al. 2019).

Sclerotinia sclerotiorum partitivirus 1 (SsPV1; family *Partitiviridae*, isometric bisegmented dsRNA viruses) reduces growth and virulence when infecting both *S. sclerotiorum* and *B. cinerea* (Xiao et al. 2014). SsPV1‐infected *B. cinerea* strains failed to develop germ tube formation, rendering infections lethal. Both species belong to the *Sclerotiniaceae* family, share ecological niches and often co‐infect host plants. Cross‐infections of viruses between *B. cinerea* and *S. sclerotiorum* are common (Hao et al. 2018; Hai et al. 2022; Deng et al. 2022; Duan et al. 2024), yet whether this is due to their close evolutionary relationship or via transmission during co‐infections of the same plant remains unknown. Viral transmission across fungal‐fungal or fungal‐plant interfaces during co‐infections is a naturally occurring phenomenon (Andika et al. 2023). SsHV2 and SsPV1 demonstrate why true host ranges and off‐target effects in other pathogenic fungi are key to understanding before commercialising mycovirus applications.

Sclerotinia sclerotiorum mycoreovirus 4 (SsMYRV4; family *Reoviridae*, isometric multisegmented dsRNA viruses) also induces hypovirulence by reducing growth and sclerogenesis in *S. sclerotiorum*. Wu et al. (2017) found host heterokaryon incompatibility (*het*), G‐protein subunit and reactive oxygen species (ROS)‐related genes were all downregulated during SsMYRV4 infections. This resulted in the suppression of non‐self recognition between incompatible hyphae and horizontal transmission of SsMYRV4 and any co‐infecting heterologous viruses into previously incompatible strains on *B. napus* . For example, Sclerotinia sclerotiorum debilitation associated RNA virus (SsDRV; family *Alphaflexiviridae*, filamentous monosegmented ssRNA viruses) infections, alongside their hypovirulent phenotype, were transmitted between previously incompatible strains by co‐infecting SsDRV with SsMYRV4 (Xie et al. 2006). In addition to SsMYRV4's hypovirulent symptoms, it can potentially be used as a ‘helper virus' to enable transmission of other debilitating mycoviruses and should be tested under field conditions.

### 2.2 Phoma Stem Canker

Phoma stem canker (PSC) or blackleg, is caused by the ascomycetes *Leptosphaeria biglobosa* and *Leptosphaeria maculans* (anomorph *Phoma lingam*; family: *Leptosphaeriaceae*). PSC devastates brassicas globally and is considered one of three major diseases of oilseed rape alongside SSR and clubroot (Zheng et al. 2020). *Leptosphaeria* develop pseudothecia (sexual fruiting bodies) on plant debris from the previous growing season and release ascospores once mature. *Leptosphaeria* infections begin as grey leaf spots, spread asymptomatically through petioles into the stems to form cankers later in the growing season. Earlier stem base cankers, predominantly caused by *L. maculans*, are responsible for most of the yield losses (Fitt et al. 2006). Some R genes provide effective resistance to *L. maculans* in resistant *B. napus* cultivars (Mitrousia et al. 2018). In the UK, where phoma stem canker is estimated to cost £60–100 million annually (www.cropmonitor.co.uk), management is becoming increasingly reliant on azole‐based fungicides (Ridley et al. 2022). PSC remains a persistent disease and is estimated to cause over £1 billion global oilseed rape losses every year (Zhang et al. 2014).

Only four viral families have been discovered in *L. biglobosa* (Table 2) along with several uncharacterised mycoviruses and only a single mycovirus belonging to the *Endornaviridae* family has been found in *L. maculans* (unpublished). As *L. maculans* is responsible for most of the economic damage, discovery of novel hypovirulence‐associated mycoviruses would have considerable potential impact. Mycoviruses infecting *Leptosphaeria* remain poorly understood and yet, retain at least one promising candidate for PSC control.

Leptosphaeria biglobosa quadrivirus‐1 (LbQV‐1; family *Quadriviridae*, isometric quadripartite dsRNA viruses) provides an alternative, hypervirulence‐based form of disease control. LbQV‐1 increases growth, virulence, sporulation and pigmentation upon infecting *L. biglobosa*, commonly associated with the less severe upper stem lesions (Shah et al. 2018). Upon further investigation, Shah et al. (2020) demonstrated LbQV‐1‐infected *L*. *biglobosa* protected *B. napus* against subsequent *L. maculans* infections, shown by reduced lesions in systemic leaves. The extent of LbQV‐1‐mediated resistance to *L. maculans* and other pathogens should be fully explored in field trials. Earlier studies evidence similar protective qualities of ‘non‐aggressive’ *L. biglobosa* isolates under field conditions; however, it is unknown if this was due to mycovirus infections (Mahuku et al. 1996; Liu et al. 2006; Thomas et al. 2009). LbQV‐1‐infected *L. biglobosa* infections severely altered the *B. napus* transcriptome when compared to virus‐free *L. biglobosa* infection plants. Notably, pathogenesis‐related (PR) and chitinase genes were upregulated (Shah et al. 2020), suggesting key systemic acquired resistance (SAR)‐related immune pathways are involved in LbQV‐1‐mediated oilseed rape resistance.

*Leptosphaeria* also hosts Leptosphaeria biglobosa botybirnairus‐1 (LbBV‐1; family *Botybirnaviridae*, isometric bisegmented dsRNA viruses). Despite LbBV‐1 infections in *L. biglobosa* being asymptomatic, LbBV‐1‐infected *B. cinerea* displayed lower virulence. Interestingly, LbBV‐1 can cross‐transmit between *L. biglobosa* and *B. cinerea* more efficiently during *B. napus* co‐infections (18.8%) compared to co‐cultures (4.6%). Screening of *L. biglobosa* and *B. cinerea* field isolates also uncovered several families of *Botrytis* viruses cross‐infecting *Leptosphaeria* (Deng et al. 2022). Thus, in pathogens such as *Leptosphaeria*, more emphasis should be placed upon investigating cross‐infections of mycoviruses from generalist pathogens that share infection niches.

### 2.3 Grey Mould

The ascomycete *Botrytis cinerea* (family *Sclerotiniaceae*) causes grey mould and notoriously infects over 1000 plant species (Elad et al. 2016). Grey mould control is estimated to cost over €1 billion annually (Dean et al. 2012). *B. cinerea* is an opportunistic, necrotrophic pathogen which infects dead or decaying tissue of many brassicas. Grey mould infections often begin as brown‐grey lesions and spread into the locally surrounding tissue. In some cases, *B. cinerea* may colonise stems or seed pods, thus reducing yield quality, yet it is not considered a significant threat to oilseed rape production (Bayer Crop Science 2026; Zheng et al. 2020). Despite limited direct impact on oilseed rape plants, viruses of *B. cinerea* and other generalist pathogens have been widely explored (Khalifa et al. 2024). Generalist pathogens may provide viruses capable of infecting unconventional hosts and inducing debilitating phenotypes in high impact oilseed rape pathogens. Furthermore, *B. cinerea* shares ecological niches with many other pathogens and its role as a ‘bridge pathogen’ enabling cross‐transmission of viruses among evolutionarily and ecologically distant pathogens is becoming increasingly apparent (Deng et al. 2003, 2022).

To date, six out of 22 mycovirus families discovered in *B. cinerea* display promise for biocontrol (Table 2). dsRNA screening revealed a high abundance of mycoviruses in *B. cinerea* populations (Ruiz‐Padilla et al. 2021). Duan et al. (2025) demonstrated 404 out of 406 strains harboured mycovirus infections, with 86% of strains containing over six infections and individual strains holding up to 25 infections simultaneously.

The ssDNA mycovirus Botrytis gemydayirivirus 1 (BGDaV1) was discovered alongside BGDaV2 in a non‐pathogenic grey mould isolate. Belonging to *Genomoviridae*, BGDaV1 induces hypovirulence in *B. cinerea* and is infectious as extracellular viral particles (Khalifa and MacDiarmid 2021). Provisional experiments suggest BGDaV1 may be able to partially protect *B. napus* against future *B. cinerea* infection and should be explored further. Interestingly, the BGDaV1 titre remained very low in naturally infected isolates due to host RNA silencing mechanisms. RNA silencing plays a key role in antimycoviral defence (Donaire and Ayllón 2017) and suppression of antiviral host machinery is a common mechanism employed by mycoviruses to facilitate infection (Rodriguez Coy et al. 2022; Lalany 2025). BGDaV1 holds many of the attributes required for biocontrol and methods to suppress RNA silencing in *B. cinerea* warrant further investigation.

Discovered on tomato plants, the *B. cinerea* strain QT5‐19 was infected by Botrytis cinerea partitivirus 2 (BcPV2) which induced defective conidiogenesis and sclerogenesis (Kamaruzzaman et al. 2019). QT5‐19 outcompeted virulent strains of *B. cinerea* and *S. sclerotiorum* isolates by producing antifungal volatile organic compounds (VOCs). This was not due to BcPV2 infection and the presence of other mycovirus infections should be investigated. Furthermore, QT5‐19 stimulated plant growth through upregulation of hormonal and metabolic pathways (Kamaruzzaman et al. 2020, 2021) and QT5‐19's debilitating phenotype was consistent across many crops including oilseed rape. As QT5‐19 possesses many qualities desired in biocontrols/biostimulants, its viability in a wider range of brassicas should be explored.

### 2.4 Alternaria Black Spot

The ascomycete genus of *Alternaria* (family *Pleosporaceae*) contains pathogenic species known to infect a wide variety of crops (Singh Saharan et al. 2016). *Alternaria brassicae* and *A. brassiciola* are the main causal agents of Alternaria black spot (ABS) in brassicaceous crops, yet other *Alternaria* species may also infect *B. napus* (Al‐lami et al. 2020). *Alternaria* can infect most tissue types and in edible brassicas, leaf spots spoil commercial produce. Infections produce black spots on seed pods or brown spots surrounded by yellow concentric rings on leaves. *Alternaria* infections may then develop into more severe necrotic lesions and cause root rot, chlorosis and poor seed germination. If left unchecked, *Alternaria* infections spread and shrivel seeds, reducing oil content thus lowering yields up to 70% (Degenhardt et al. 1974). Discovering effective resistance to *A. brassicae* capable of genetic transfer in breeding programmes has proven difficult (Meena et al. 2010).

In total, six out of the 16 mycovirus families discovered in *Alternaria* exhibit BCA potential (Table 2). Screening of *A. alternata* isolates revealed high prevalence of mycoviruses (Tian et al. 2022) and hypovirulence‐associated infections were first discovered in *Alternaria* in 2009 (Aoki et al. 2009). Members of *Endornaviridae* (Shang et al. 2015), *Fusariviridae* (Zhong et al. 2016) and *Mitoviridae* (Chen et al. 2017) have been discovered in *A. brassiciola*, but appear to have no biological impact. Thus, screening hypovirulent *A. brassicae* /*A. brassiciola* isolates should remain the current priority.

Mycoviruses have been found to induce hypovirulence in other *Alternaria* species. *Alternaria alternata* chrysovirus 1 (AaCV1; family *Chrysoviridae*, isometric multi‐segmented dsRNA viruses) mediates hypovirulent phenotypes differently in both *A. alternata* and *A. tenuissima* . AaCV1 inhibits growth and reduces production of the mycotoxin alternariol in *A. alternata* . AaCV1 infection was discovered alongside *Alternaria alternata* magoulivirus 1 (AaMV1; family *Botourmiaviridae*, capsidless monosegmented ssRNA viruses). AaMV1 did not impact virulence, but co‐infection increased the rate of AaCV1 horizontal transmission (Li et al. 2022). In *A. tenuissima* , AaCV1 attenuates growth, lowers sporulation and increases sensitivity to two antifungal agents, difenoconazole and tebuconazole (Ma et al. 2020). Zhang et al. (2025) further determined that AaCV1/AaMV1 infections utilize small RNAs to mediate fungal virulence. AaCV1 and AaMV1 demonstrate the potential of co‐infections to enhance mycovirus‐mediated disease control in a multi‐faceted manner. We suggest the mechanisms of AaMV1‐mediated elevated transmission should be fully investigated.

### 2.5 Other Relevant Diseases of Oilseed Rape

*Rhizoctonia solani* and *Fusarium* are generalist pathogens known to infect and impact yields of oilseed rape (Verma 1996; Jedryczka et al. 2002). Most *R. solani* anastomosis groups (AG) threaten brassicas; AG2‐1 contains the most virulent strains to *B. napus* (Abdoulaye et al. 2019; Sims et al. 2023). *Fusarium* reduces oilseed rape yields by wilt (*Fusarium oxysporum f*.sp. *conglutinans*) (Gaetán 2005) or blight (*Fusarium* spp.; Chen et al. 2014). Research into both pathogens has revealed extensive mycoviral diversity (not covered in this review; Umer et al. 2023; Zhang 2022). As *R. solani* and *Fusarium* may act as bridge pathogens in a similar manner to *B. cinerea* , the potential of their mycoviruses to cross‐infect and modulate phenotypes of other oilseed rape pathogens should also be investigated.

Verticillium stem stripe is caused by the soil‐borne fungus *Verticillium longisporum* and impacts oilseed rape cultivation mainly in Europe (Gladders et al. 2011) and Canada (CFIA 2015). Field studies define the impact of *V. longisporum* to vary considerably (Dunker et al. 2008), especially when combined with abiotic stress (AHDB 2026). Hypovirulence associated with mycovirus infections of *V. dahlie*, a generalist *Verticillium* pathotype, has been discovered (Gao et al. 2025), yet the presence of mycoviruses in *V. longisporum* remains completely unexplored. Recent research into manipulating the endocytic pathway for exogenous RNA uptake highlights the opportunity to develop mycovirus‐based applications in *Verticillium* (Liu et al. 2025).

In Europe, white leaf spot (*Pseudocercosporella capsellae*) and light leaf spot (*Pyrenopeziza brassicae*) are two emerging pathogens of oilseed rape (Williams 2010; Su et al. 1998). Both *P. capsellae* and *P. brassicae* infect most brassicaceous crops. Cultural methods only provide effective white leaf spot control in combination with favourable weather conditions and there is a lack of effective biocontrol solutions (Gunasinghe et al. 2020). *P. brassicae's* asymptomatic early infection stage makes disease monitoring difficult and reduces yields by up to 1 t/ha in the UK (AHDB 2026). No mycoviruses have been discovered in either pathogen and current research should prioritise screening hypovirulent strains.

## 3 Mycoviruses Associated With the Pests of Oilseed Rape: EPF

The threat of pests to oilseed rape production is typically greater than diseases in most cultivating regions. Pest such as flea beetles, beetles, weevils, mites, moths, aphids and slugs are represented by many individual species which vary greatly in geographic distribution (Zheng et al. 2020). For example, cabbage stem flea beetles (CSFB; *Psylliodes chrysocephala*) devastate oilseed rape fields across Europe yet do not concern growers in Australia (Gu et al. 2007). Flea beetles (*Phyllotreta* spp.), namely the striped flea beetle (SFB; *Phyllotreta striolata* ) and the crucifer flea beetle (CFB; *Phyllotreta cruciferae* ), replace CSFB in prominence in China and North America (Li et al. 2024). Similar methods make up pest and disease management, yet effective pest solutions remain scarce and successful control relies heavily on insecticides. Recent restrictions on insecticides have made pest management increasingly challenging (Blake 2018). Some biological alternatives exist, however efficacy varies significantly under field conditions (Orr 1988).

**FIGURE 2:**
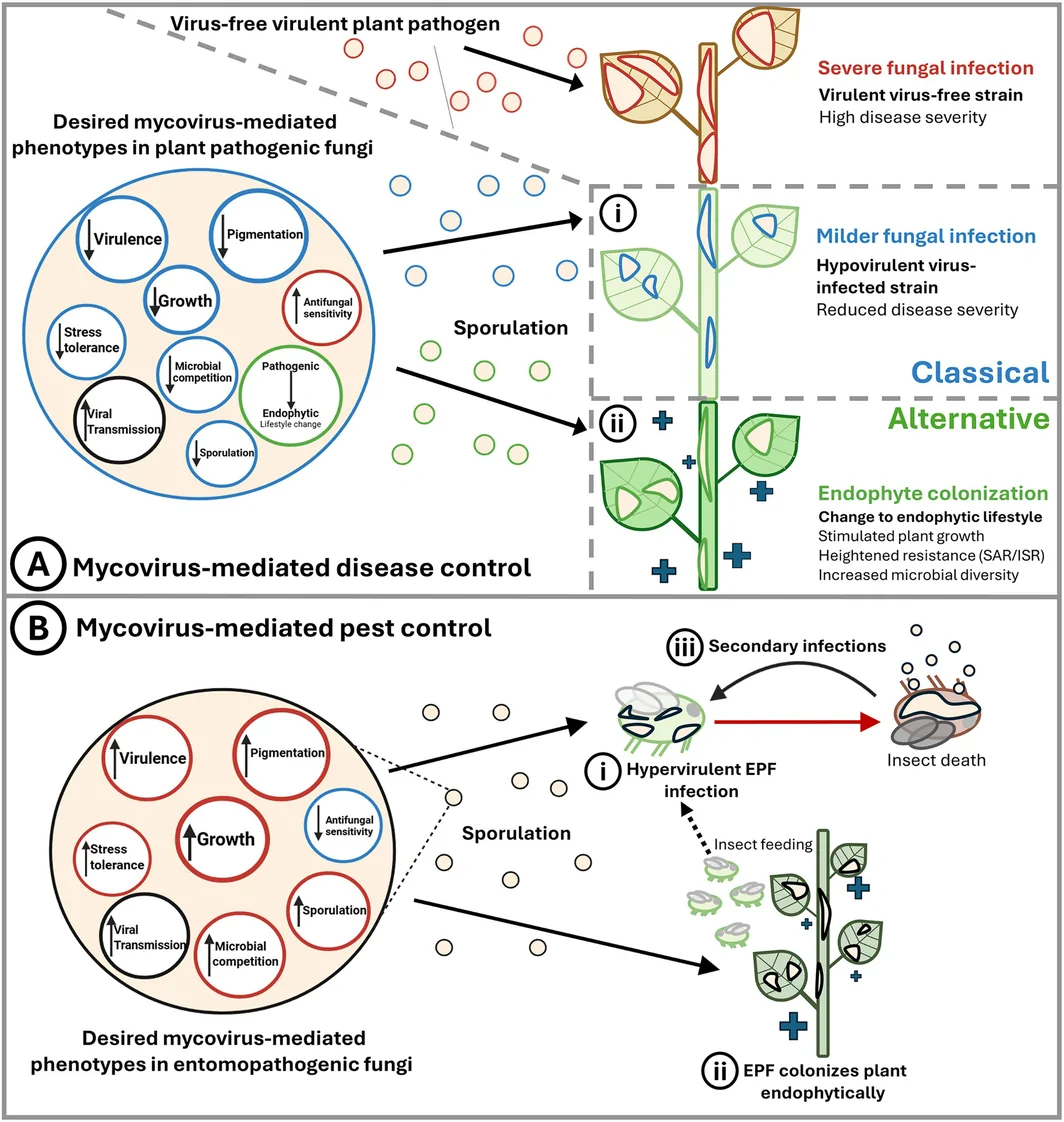
The basis of mycovirus‐mediated biocontrol. (A) Mycovirus‐mediated disease control. Mycoviruses may induce a range of desired (i), hypovirulent or (ii) endophytic phenotypes in plant pathogenic fungi. (i) Hypovirulent mycovirus infections will transmit through a fungal population and compete with virulent pathogen strains to reduce disease severity. (ii) Mycovirus‐induced endophytes may stimulate plant growth, heighten disease resistance and increase plant‐associated microbial diversity, thus reducing overall disease severity. (B) Mycovirus‐mediated pest control. Mycoviruses may induce hypervirulent phenotypes upon infecting entomopathogenic fungi (EPF) and augment their biocontrol capabilities. Hypervirulent EPFs both (i) infect and kill insects and (ii) colonise plants as endophytes with elevated effectiveness. As endophytes, EPFs may stimulate plant growth, induce pest/disease resistance and transmit to insects during feeding. (iii) EPFs sporulate after insect death and lead to secondary insect infections.

EPF infect and cause disease in all major insect types (Pu et al. 2025; Sabbahi and Hock 2024; Duarte et al. 2016; Lee et al. 2015). Infection strategies vary, yet many EPF penetrate the insect cuticle, colonise the host tissue and circulatory system and release toxins which can result in death within 3–7 days. Upon death, EPF often initiate conidiation to facilitate secondary infections (Shah and Pell 2003). In addition to prohibiting herbivorous insects, some EPF species exhibit saprophytic and endophytic lifestyles (Tall and Meyling 2018). These species enable transmission when insects feed on a host plant, thus making EPF extremely attractive BCAs. Mycovirus‐induced hypervirulence in EPFs can augment their effectiveness as pest control (Figure 2b) and is an emerging area of research.

### 3.1 Beauveria Bassiana

The ascomycete *Beauveria bassiana* (family *Cordycipitaceae*) can infect over 700 insect species and is considered a generalist mycoinsecticide. Virulence of *B. bassiana* isolates vary significantly among insects and environmental conditions (Uma Devi et al. 2008), yet commercial *B. bassiana* products can be effective at controlling most insect orders under field conditions (Clark et al. 1968; Wraight and Ramos 2002; Zimmermann 2007; Pordilla et al. 2017). *B. bassiana* colonises plants endophytically and improves yields of many crops (Wagner and Lewis 2000; Deb et al. 2022) including oilseed rape (Vidal and Jaber 2015; Muola et al. 2024). As an endophyte, *B. bassiana* promotes plant growth, increases microbial diversity and heightens resistance against a range of biotic threats (Chang et al. 2021; Sui et al. 2023).

In total, two out of six mycovirus families discovered in *B. bassiana* present biocontrol potential (Table 2). Polymycoviruses (family *Polymycoviridae*, unconventionally encapsidated multisegmented dsRNA viruses) show promise for disease control in plants, humans and insects (Zhai et al. 2016; Kanhayuwa et al. 2015; Kotta‐Loizou and Coutts 2017). Beauveria bassiana polymycoviruses (BbPmV)‐1,2 and 3 were first discovered in 2017. BbPmV‐1, alongside the co‐infection of an unclassified virus Beauveria bassiana non‐segmented virus‐1 (BbNV‐1; family *Amalgaviridae*, capsidless monosegmented dsRNA viruses), induced mild hypervirulence. Hypervirulent *B. bassiana* isolates reduced the survival of greater wax moth larvae (*Galleria mellonella*) when compared to virus‐free isolates (Kotta‐Loizou and Coutts 2017), possibly due to the upregulation of virulence factors such as the mycotoxin beauvericin (Filippou et al. 2025). Overproduction of mycotoxins during insect infections enhances EPF virulence (Altinok et al. 2019). Mycotoxins also regulate virulence in phytopathogens, are natural contaminants in edible crops and their cytotoxic effects pose serious health threats if consumed (Hasuda and Bracarense 2024). Little is known regarding the role of mycotoxins and how mycoviruses influence their production, especially during EPF endophytism. During co‐infection with a partitivirus, BbPmV‐1 additionally enhances drought, osmotic and UV stress tolerance (Rueda‐Maíllo et al. 2025). Further research uncovered the ability of both BbPmV‐1 and BbPmV‐3 to increase conidiation and pigmentation upon infecting *B. bassiana* (Filippou et al. 2021).

BbPmV‐4 induces similar hypervirulent phenotypes and upregulates host virulence factors (Kang et al. 2021, 2023). Polyketide synthase (PKS) synthesises secondary metabolites that inhibit insect antimicrobial compounds and has elevated expression during BbPmV‐4 infections. A closely related viral strain BbPmV4‐2, containing four additional dsRNA genomic segments, also increases host virulence alongside conidiation, heat and UV tolerance. Functional analysis suggests a BbPmV4‐2 protein, P5, mediates these phenotypes through interactions with host proteins regulating cell membrane and ubiquitination (Shi et al. 2025). BbPmV‐4(−2)‐mediated transcriptomic changes enhance fungal virulence and stress management, suppress insect immune responses and mediate insect cell toxicity. Thus, multiple *Beauveria* polymycoviruses demonstrate biocontrol potential and future investigations should delve into their impact on *B. bassiana's* endophytic lifestyle.

In *Beauveria* spp. both polymycoviruses and chrysoviruses exhibit stable interspecific transmission during insect co‐infections (Ning et al. 2022). Chrysoviruses also mediate virulence in *B. bassiana*. PKS, beauvericin and virulence‐related genes upregulated during BbPmV‐4 infections are downregulated during Beauveria bassiana chrysovirus‐2 (BbCV‐2) infections, resulting in hypovirulence and improved insect performance (Zhang et al. 2023). Conversely, BbCV‐2 infections enhanced fungal growth and conidiation when *B. bassiana* grew endophytically, enabling better colonisation of its plant host. Furthermore, BbCV‐2 upregulated cell wall‐degrading enzymes and antimicrobial secondary metabolites in *B. bassiana*, enabling it to outcompete *S. sclerotiorum* and *B. cinerea in planta* (Sui et al. 2024). Thus, BbCV‐2 has the potential to both enhance and hinder different beneficial attributes of *B. bassiana*. Future research should uncover how the molecular mechanisms behind BbCV‐2‐mediated phenotypes differ in fungal‐insect and fungal‐plant interactions. Similar investigations have uncovered how individual mycoviral proteins mediate host phenotypes (Bormann et al. 2018; Takahashi‐Nakaguchi et al. 2019; Shi et al. 2025).

Another recently discovered dsRNA mycovirus, Beauveria bassiana orthocurvulavirus 1 (BbOCuV1; unclassified; Xu et al. 2024), has demonstrated potential to enhance *B. bassiana's* biocontrol capabilities. BbOCuV1 increased growth, sporulation and biomass production upon infection. Furthermore, BbOCuV1‐infected lines inhibited two phytopathogens, *S. sclerotiorum* and *Fusarium asiaticum*, in vitro and in planta post‐endophytic colonisation (Guo et al. 2026).

### 3.2 Metarhizium spp.

The ascomycete genus *Metarhizium* (family *Clavicipitaceae*) contains many pathogenic and endophytic species. Despite their narrower host ranges, *Metarhizium* persist in soil for extended periods and are sold as effective commercial insecticides (Chandwani et al. 2023). Several species control oilseed rape pests. *M. anisopliae* can infect and control diamondback moths (*Plutella xylostella*) (Batta 2013) and mustard aphids ( *Lipaphis erysimi* ; Ujjan and Shahzad 2012). *M. brunneum* promotes growth in *B. napus* , provides protection against fungal diseases such as *V. longisporum* and varies in effectiveness against insect pests (Posada‐Vergara et al. 2023). Colonisation by *M. robertsii* increases above‐ground biomass in *B. napus* but is yet to be directly linked to biocontrol (Ahmad et al. 2020).

Two of four mycovirus families discovered in *Metarhizium* demonstrate biocontrol potential (Table 2). Screening for novel mycoviruses should thus focus on the aforementioned *Metarhizium* species, in addition to other commercially used species (Yapa et al. 2025).

Partitiviruses have been discovered in several *Metarhizium* species. Discovered in an *M. flavoviride* strain infecting small brown planthoppers ( *Laodelphax striatellus* ), Metarhizium flavoviride partitivirus 1 (MfPV1) infections increased conidiation and expression of virulence factors. Mad1, encoding an adhesin protein responsible for conidia attachment onto insect cuticles, along with genes encoding cuticle‐degrading proteases and enzymes mediating insect cell toxicity, were upregulated upon MfPV1 infection. MfPV1 was also transfected into *M. anisopliae* and *M. pingshaense*, where it induced hypervirulence and elevated mortality of diamondback moths and fall armyworms ( *Spodoptera frugiperda* ; Guo et al. 2024). Another partitivirus, Metarhizium anisopliae virus RJ (MaV‐RJ), increased conidiation but reduced endochitinase secretion in *M. anisopliae* (De La Paz Giménez‐Pecci et al. 2002). Endochitinases mediate cuticle degradation and intermicrobial competition, thus impacting both *M. anisopliae's* entomopathogenic and endophytic lifestyles.

However, partitiviruses can also reduce virulence in *Metarhizium*. Metarhizium majus partitivirus 1 (MmPV1)‐infected isolates had lower conidiation and tolerance to heat shock and UV radiation when compared with virus‐free *M. majus* isolates. Interestingly, production of triterpenoids (antiviral proteases) and metarhizins (compounds associated with inhibiting proliferation of insect hemocoels) was inhibited (Wang, Yang, Shi, et al. 2023; Kikuchi et al. 2009) during MmPV1 infections and could facilitate viral propagation during insect infections. Neither change in metabolites was directly linked to an individual viral protein; however, MmPV1‐mediated transcriptional changes could be manipulated to facilitate heterologous viral infections.

Polymycoviruses have also been found to infect multiple *Metarhizium* species (Wang, Yang, Lu, and Huang 2023; He et al. 2023; Oliveira et al. 2025). Metarhizium anisopliae polymycovirus 1 (MaPmV1) infections enhanced fungal growth and conidiation yet did not impact virulence and MaPmV1 additionally lowered UV tolerance (Wang, Yang, Lu, and Huang 2023). Nevertheless, in the last 5 years, the diversity of *Metarhizium* viruses has expanded rapidly and should pave the way for other EPFs with little mycovirus exploration (Camargo et al. 2025). The genera *Cordyceps* (*Ascomycota*; family *Cordycipitaceae*), *Ophiocordyceps* (*Ascomycota*; family *Ophiocordycipitaceae*), *Isaria* (*Ascomycota*; family *Cordycipitaceae*) and *Entomophthora* (*Entomophthoramycota*; family *Entomophthoraceae*) contain many species parasitic to insects, including oilseed rape pests (Lei et al. 2021; Du et al. 2021) and remain in their infancy of mycovirus research.

## 4 Progress and Current Challenges of Mycovirus‐Based Applications

In recent years, improvement in NGS technology and an increasing frequency of screening studies have resulted in the rapid discovery of mycoviruses in important pathogenic fungi. In the field, the sequencing, classification and subsequent biological characterisation of novel viruses have yielded numerous potential candidates for biocontrol. However, many aspects of mycovirology remain poorly understood.

### 4.1 Mycovirus‐Based Applications

To reduce disease severity, mycoviruses must typically induce hypovirulence or other debilitating symptoms, upon infecting a phytopathogenic fungus. This is somewhat the ‘classical’ model for mycovirus‐based disease control (Figure 2Ai). A hypovirulent strain (e.g., CHV1‐infected *C. parasitica* ) released into the environment must outcompete virulent strains (CHV1‐free *C. parasitica* ) and reduce overall disease severity in the field. Reduced growth, sporulation and other natural barriers of mycovirus transmission (e.g., vegetative compatibility) may hinder the spread of classical hypovirulence‐based control methods under field conditions. However, as insight into the role of mycoviruses in mediating plant‐fungal interactions deepens, an ‘alternative’ form of mycovirus‐based control has emerged (Figure 2Aii). Alongside hypovirulence‐associated traits, mycovirus infections may change their host's lifestyle from pathogenic to endophytic (Qu, Zhang, et al. 2021; Zhou et al. 2021), stimulate plant growth (Zhang et al. 2020) and induce systemic resistance to subsequent pathogen attacks (Qu et al. 2020; Shah et al. 2020; Zhou et al. 2021; Wang et al. 2024). In instances of mycovirus‐induced endophytism, increased growth and sporulation may actively encourage colonisation and positively benefit the host plant. Mycovirus‐induced endophytes can thus reduce disease severity directly, by competing with virus‐free pathogenic counterparts and indirectly through elevating plant growth (biostimulant) and heightening resistance against subsequent biotic threats (biocontrol). The biostimulant/biocontrol potential of EPFs, which possess both entomopathogenic and endophytic life cycles, may also be elevated by hypervirulent mycovirus infections (Figure 2B). Hypervirulent EPFs can control insect populations more effectively whilst also improving plant health and resistance traits. Thus, both mycovirus‐induced endophytes and mycovirus‐enhanced EPFs can potentially be applied in a similar preventative manner to beneficial microbes early in the growing season. These alternative forms of mycovirus‐mediated control bypass some of the natural obstacles associated with hypovirulence‐dependent disease control.

### 4.2 The Obstacles of Mycovirus Transmission

Reduced sporulation, while typically a desired characteristic in hypovirulence‐based fungal applications, may hinder mycovirus transmission through a population. Mycoviruses spread vertically into spores and horizontally through anastomosis. Efficiency of vertical transmission depends on the specific fungus‐mycovirus combination (Buivydaitė et al. 2024), if sporulation is sexual (Deng et al. 2007) and can be up to 100% efficient (Jacquat et al. 2020; Hua et al. 2024). Interestingly, sporulation does not always directly correlate to disease severity (He et al. 2025) and effective mycovirus transmission has been demonstrated as key in successful mycovirus‐based applications (Anagnostakis 1982). Effective horizontal transmission through a population is often restricted by vegetative incompatibility. However, the widespread prevalence of ‘core viruses’, such as *Botrytis virus F*, across vegetative compatibility groups (VCGs) suggests some mycoviruses are capable of transmission regardless of compatibility (Arthur and Pearson 2014). Wu et al. (2017) demonstrated the ability of SsMYRV4 to suppress non‐self‐recognition between incompatible hyphae, enabling not only SsMYRV4, but heterologous virus transmission to previously incompatible strains of *S. sclerotiorum*. Furthermore, transmission across VCGs was more efficient under elevated proline levels in planta or through exogenous application to OSR (Hai et al. 2024). These discoveries have begun to reveal the mechanisms behind mycovirus transmission and vegetative incompatibility inhibition and will aid the next steps to manipulate mycovirus transmission under field conditions.

### 4.3 Co‐Infection Dynamics

SsMYRV4 also induces hypovirulence. Due to the wealth of research into *S. sclerotiorum*, many mycoviruses have been found to induce multiple phenotypes (Table 2), thus possessing multi‐faceted biocontrol potential. In cases where complementary phenotypes do not occur as symptoms of a single infection, co‐infections provide an alternative solution to maximise biocontrol potential in mycovirus‐infected strains. For example, AaMV1 increases heterologous virus transmission yet does not impact virulence itself. Thus, coupling AaMV1 with a debilitating mycovirus (e.g., AaCV1) amplifies biocontrol potential (Li et al. 2022). As abundance of co‐infections becomes increasingly apparent (Marzano et al. 2016), new research suggests mycoviruses mediate their co‐infecting viruses by sharing/competing for infection niches within a host. Duan et al. (2025) analysed co‐infections across a population of *B. cinerea* isolates and devised ‘co‐infection rules’, where the presence/absence of one virus was highly dependent on the presence/absence of another. When considering many mycoviruses mediate complementary or opposing phenotypes, relationships between mycoviruses within the host population should be thoroughly investigated before application. For example, virus A may induce hypovirulence but also encourage the co‐infection of a hypervirulent virus B. Thus, application of virus A would likely have little impact on disease severity across a fungal population. The analytical methods used in this study should be applied in a more focussed manner to symptomatic viruses of high impact pathogenic fungi.

### 4.4 Host Range Unpredictability

Host ranges of mycoviruses are poorly understood. In addition to mediating multiple phenotypes in their natural hosts, mycoviruses may induce different phenotypes in multiple fungal species (Deng et al. 2022; Tran et al. 2019). Many fungal pathogens share ecological niches and co‐infect plant hosts simultaneously. As the fungal‐fungal interface enables cross‐transmission of mycoviruses between co‐infecting fungi (Jia et al. 2024), understanding how the phenotypes induced upon mycoviral infection differ across each species in a mycovirus's host range is key prior to application in the field. Furthermore, transmission across the plant‐fungal interface (Andika et al. 2023) and the ability of some mycoviruses to unconventionally replicate in plants (Nerva et al. 2017; Dai et al. 2024; Wang et al. 2024) or insects (Liu et al. 2016) give rise to potential new avenues for mycoviral transmission.

### 4.5 Prospects for Engineered Vectors

Many mycoviruses share close evolutionary relationships with plant viruses and are often classified within the same viral family. Mycoviruses capable of systemic cross‐kingdom plant infections may encode movement‐like proteins with high sequential and functional similarity to plant viral movement proteins (MPs; Dai et al. 2024; Wang et al. 2024). MP expression, whether native to the mycovirus or transgenic (Bian et al. 2020), can elevate infection efficiency, yet is not sufficient for some cross‐kingdom infections (Bono et al. 2026). How mycoviruses can sustain permanent infections in insect hosts is largely unknown. Understanding how mycoviruses can (i), infect unconventional hosts systemically and (ii), replicate in unconventional host cells may enable us to manipulate plants/insects into vectors for mycovirus‐based applications, in which existing viral technology could be harnessed.

Viral vectors are commonly used in both gene therapy (Lundstrom 2018) and plant bioengineering (Velásquez et al. 2009). Universal donors are central to these technologies and permit insertion of transgenic material specific to the application. Some regard the development of a universal mycovirus donor as one of the holy grails of mycovirology (Xie and Jiang 2014). Understanding how proteins encoded by previously mentioned mycoviruses capable of overcoming vegetative incompatibility, cross‐kingdom transmission barriers and other desirable phenotypes will be key to developing a universal donor. These mycoviral genomes may potentially provide vital transgenic sequences for future mycoviral engineering.

The recent discovery of a yadokavirus in *S. sclerotiorum*, which can hijack heterologous viral capsid proteins to facilitate self‐replication, illustrates a natural example of ‘donor‐like’ behaviour. Sclerotinia sclerotiorum yadokarivirus 1 (SsYkV1; family *Yadokaviridae*, capsidless, non‐segmented ssRNA viruses) replicates, likely in a similar manner to dsRNA viruses, whilst trans‐encapsidated by Sclerotinia sclerotiorum botybirnavirus 3 (SsBV3) capsid proteins during co‐infections (Jia et al. 2022). Another member of Y*adokaviridae* is also capable of hijacking heterologous capsid proteins for self‐replication (Zhang et al. 2016). The hijacking mechanism and the extent to which yadokaviruses may exploit other heterologous viruses in other co‐infection scenarios remain poorly understood. SsYkV1 has little biological effect upon infection and remains a promising candidate in the development of a universal mycovirus donor.

### 4.6 Phenotypic Instability and Field Reproducibility

Currently, lack of commercial viability limits the integration of mycovirus‐based solutions into agricultural management programs. Only SsHADV‐1‐infected strains have been extensively proven to reduce field disease severity and increase yields (Qu et al. 2020; Tian et al. 2020; Chen et al. 2026) whilst other promising mycovirus‐infected strains lack field data. Furthermore, there is little knowledge of how dynamic abiotic factors impact mycoviruses in the environment over time. Mycovirus infections and their respective phenotypes can be unstable over long periods (Zamora‐Ballesteros et al. 2021) and even under controlled growth conditions, strains infected by the same mycovirus may display severely different symptoms (Zhang et al. 2012). Thus, when considering abiotic stresses are typically used to aid the curing of mycovirus‐infected isolates, it is reasonable to predict that changing environmental conditions may elevate mycovirus instability within host populations. It is therefore imperative we understand the mechanisms underlying mycovirus transmission and co‐infection and how they change under field conditions during the development of mycovirus‐based BCAs.

## 5 Concluding Remarks

Here, we have reviewed mycoviruses with potential to control pests and diseases of oilseed rape and discussed current challenges facing mycovirus‐based applications. Mycovirologists should prioritise screening hypovirulent isolates in pathogens where mycovirology is largely unexplored. For pathogens rich in promising mycovirus candidates for biocontrol, the current challenge is testing applications under field conditions as mycovirus‐mediated phenotypes can be unstable over time. Poor understanding of mycovirus transmission within fungal populations in combination with well‐established DNA‐based applications in agriculture, results in exogenous mycovirus applications (e.g., SsHADV‐1) remaining more promising than RNA mycovirus applications. Nevertheless, recent research into mycovirus cross‐transmission and advancements in dsRNA applications in agriculture (McLoughlin et al. 2018) provide new hope for RNA mycovirus applications. Mycovirus‐based BCAs are not yet commercially viable, but uncovering the mechanisms behind mycovirus‐mediated phenotypes like vegetative incompatibility and antifungal sensitivity would accelerate integration into IPMs.

## Author Contributions

H.J.A. wrote the first draft, created all figures and tables and compiled the references. I.K.‐L. edited the manuscript, revised figures and tables and aided with conceptualisation. Y‐.J.H. edited the manuscript and revised figures. R.H.A.C. edited the manuscript. All authors have read and agreed to the published version of the manuscript.

## Disclosure

No generative AI tools were used to create this manuscript.

## Conflicts of Interest

The authors declare no conflicts of interest.

## Data Availability

Data sharing is not applicable to this article as no datasets were generated or analysed during the current study.
